# Trastuzumab emtansine delays and overcomes resistance to the third-generation EGFR-TKI osimertinib in NSCLC EGFR mutated cell lines

**DOI:** 10.1186/s13046-017-0653-7

**Published:** 2017-12-04

**Authors:** Silvia La Monica, Daniele Cretella, Mara Bonelli, Claudia Fumarola, Andrea Cavazzoni, Graziana Digiacomo, Lisa Flammini, Elisabetta Barocelli, Roberta Minari, Nadia Naldi, Pier Giorgio Petronini, Marcello Tiseo, Roberta Alfieri

**Affiliations:** 1 Department of Medicine and Surgery, University of Parma, Parma, Italy; 2 Food and Drug Department, University of Parma, Parma, Italy; 3 Division of Medical Oncology, University Hospital of Parma, Parma, Italy

**Keywords:** NSCLC, EGFR, Osimertinib, T-DM1, TKI-resistance

## Abstract

**Background:**

Osimertinib is a third-generation EGFR-TKI with a high selective potency against T790M-mutant NSCLC patients. Considering that osimertinib can lead to enhanced HER-2 expression on cell surface and HER-2 overexpression is a mechanism of resistance to osimertinib, this study was addressed to investigate the potential of combining osimertinib with trastuzumab emtansine (T-DM1) in order to improve the efficacy of osimertinib and delay or overcome resistance in NSCLC cell lines with EGFR activating mutation and with T790M mutation or HER-2 amplification.

**Methods:**

The effects of osimertinib combined with T-DM1 on cell proliferation, cell cycle, cell death, antibody-dependent cell-mediated cytotoxicity (ADCC), and acquisition of osimertinib resistance was investigated in PC9, PC9-T790M and H1975 cell lines. The potential of overcoming osimertinib resistance with T-DM1 was tested in a PC9/HER2c1 xenograft model.

**Results:**

T-DM1 exerted an additive effect when combined with osimertinib in terms of inhibition of cell proliferation, cell death and ADCC induction in PC9, PC9-T790M and H1975 cell lines. Combining osimertinib and T-DM1 using different schedules in long-term growth experiments revealed that the appearance of osimertinib-resistance was prevented in PC9-T790M and delayed in H1975 cells when the two drugs were given together. By contrast, when osimertinib was followed by T-DM1 an antagonistic effect was observed on cell proliferation, cell death and resistance acquisition. In xenograft models, we demonstrated that HER-2 amplification was associated with osimertinib-resistance and that T-DM1 co-administration is a potential strategy to overcome this resistance.

**Conclusions:**

Our data suggest that concomitant treatment with osimertinib and T-DM1 may be a promising therapeutic strategy for EGFR-mutant NSCLC.

## Background

In patients with advanced Non-Small Cell Lung Cancer (NSCLC) harbouring Epidermal Growth Factor Receptor (EGFR) activating mutations, disease progression occurs after a median of 10–12 months of treatment with EGFR-Tyrosine Kinase Inhibitors (TKIs), such as gefitinib, erlotinib, and afatinib. The acquisition of the EGFR-T790M secondary mutation is the main mechanism of acquired resistance, occurring in 50–60% of the cases [1, 2].

Third-generation EGFR-TKIs, such as osimertinib, are active against both EGFR sensitizing mutant and T790M resistant tumors [3]. Osimertinib represents the new standard of care in the treatment of T790M-positive NSCLC patients resistant to previous generation TKIs [4]. However, also for this agent acquired resistance was expected and some mechanisms have been recently identified [5–7]; in particular, a new C797S mutation in exon 20 of *EGFR* gene was found as sufficient to promote resistance to osimertinib [8]. Other mechanisms include EGFR L718Q mutation [5], MET amplification [9], BRAF V600E mutation [10], and transformation to small-cell carcinoma [11]. Initial data showed that T790M mutation occurs in up to 80% of previously untreated EGFR mutated NSCLCs, suggesting that the presence of *de novo *resistant clones may be more common than previously appreciated [12], and providing evidence to support osimertinib as a potentially better first-line option compared to the currently approved EGFR-TKIs. Emerging preclinical and clinical evidence supports a scenario whereby the selective pressure imposed by the chronic exposure to targeted agents leads to the expansion of pre-existing cell clones carrying specific genetic alterations, which may ultimately become dominant. Previous studies have mainly focused on the development of strategies to overcome specific mechanisms of resistance emerging after treatment with EGFR-TKI monotherapy. However, implementing this approach clinically may be difficult given the wide variety of the identified resistance mechanisms, also for osimertinib. A more feasible,and potentially successful strategy may be to identify a combination treatment approach that prevents the occurrence of more than one resistance mechanism [6]. The good safety profile of osimertinib would permit combinations with other drugs in a tolerable fashion in clinic. Our previous data provide a rationale for combining first-generation EGFR-TKIs with monoclonal antibodies or chemotherapy [13–15]. Despite significant advances in the knowledge about osimertinib therapy, the effect of this drug in combination with monoclonal antibodies targeting HER family has not been investigated yet.

T-DM1, trastuzumab emtansine, is an antibody-drug conjugate composed by the microtubule polymerization inhibitor DM1 (derivative of maytansine) linked with a stable thioether linker to trastuzumab targeting HER-2 receptors [16]. T-DM1 was approved by FDA as a treatment for HER-2 positive pre-treated breast cancers and has been also evaluated as first-line therapy [17]. HER-2 represents a relatively new therapeutic target for NSCLC and T-DM1 is currently being tested in a phase II study in NSCLC patients with HER-2 overexpression (NCT02289833) [18]. Moreover, HER-2 amplification has been identified as a mechanism of osimertinib acquired resistance in a patient treated with osimertinib [9], providing evidence for targeting HER-2 in this clinical setting. Interestingly, HER-2 amplification occurred together with the loss of EGFR T790M mutation. Similar findings were observed by Oxnard et al. in 2 of 40 patients treated with osimertinib [19]. In addition, in vitro models provide functional evidence that HER-2 amplification may also induce innate resistance to osimertinib, confirming the clinical observations [20].

In this study we explored the potential of combining osimertinib with T-DM1 in order to improve the efficacy of third-generation EGFR-TKI and to delay or overcome resistance in NSCLC cell lines carrying EGFR activating mutation and T790M mutation or HER-2 amplification.

## Methods

### Cell culture

PC9 cells, harboring an in-frame deletion in exon 19 of EGFR gene, was kindly provided by Dr. P. Jänne (Dana-Farber Cancer Institute, Boston, MA). PC9-T790M cells were generated in our lab by exposing PC9 cells to increasing concentrations of gefitinib, and exhibited *EGFR*-T790M secondary mutation. This cell clone has been cultured in the presence of gefitinib 1 μM to maintain a selection pressure during in vitro propagation [15]. Gefitinib was removed from medium 24 h before exposing PC9-T790M to osimertinib. The PC9/HER2c1, obtained by stable transfection of PC9 cells with a HER-2 expression vector, was kindly provided by Dr. William Pao (Vanderbilt-Ingram Cancer Center, Nashville, TN). H1975 cells were from American Type Culture Collection (ATCC, Manassas, VA). Cells were cultured as recommended, regularly checked for mycoplasma contamination, and maintained under standard cell culture conditions at 37 °C in a water-saturated atmosphere of 5% CO_2_ in air.

### Drug treatment

Osimertinib was provided by AstraZeneca (Milan, Italy). T-DM1 was supplied from the inpatient pharmacy of University Hospital of Parma. Osimertinib was dissolved in DMSO (Sigma, ST Louis, MO), while T-DM1 was dissolved in 0.9% sodium chloride and diluted in fresh medium before use. Final DMSO concentration in medium never exceeded 0.1% (*v*/v) and equal amounts of the solvent were added to control cells.

### Analysis of cell proliferation, cell death and cell cycle

Cell number, cell viability and cell death were evaluated as previously described [21]. Distribution of the cells in the cell cycle was determined by propidium iodide (PI) staining and flow cytometry analysis as described elsewhere [22]. The nature of interaction between osimertinib and T-DM1 was calculated using the Bliss additivity model as previously described [23]. A theoretical dose-response curve was calculated for combined inhibition using the equation EBliss = EA + EB - EA * EB, where EA and EB are the percent of inhibition versus control obtained by osimertinib (A) and T-DM1(B) alone and the E Bliss is the percent of inhibition that would be expected if the combination was exactly additive. If the combination effect is higher than the expected value EBliss the interaction is synergistic, while if the effect is lower, the interaction is antagonistic. Otherwise, the effect is additive and there is no interaction between drugs.

### Spheroid generation

Spheroids were generated using LIPIDURE®-COAT PLATE A-U96 (NOF Corporation, Tokyo, Japan) as previously described [24]. Briefly, 500 cells were seeded and after 2 days (T_0_) the spheroids were treated with drugs for further 6 days. The effect of the drugs was evaluated in term of volume changes using the Nikon Eclipse E400 Microscope with digital Net camera. The volume of spheroids was measured [D = (Dmax + Dmin)/2; V = 4/3π(D/2)^3^] with ImageJ software and the Fold Increase (FI) index was calculated as the ratio between the spheroid volume after 6 days and the volume at T_0_.

### Flow cytometry

One million of NSCLC cells were incubated, for one hour at room temperature, with Isotype control Monoclonal Mouse IgG1/R-PE (Ancell IRP, Bayport, MN), mouse anti-Human HER-2/PE or mouse anti-Human EGFR/PE (BD Biosciences, San Josè CA) to determine EGFR or HER-2 protein membrane levels as previously described [14]. The analysis was performed using an EPICS-XL flow cytometer. Mean fluorescence intensity (MFI) values were converted in Molecules of Equivalent Fluorochrome (MEF) using the FluoroSpheres 6-Peak Kit (Dako, CA).

### Long-term culture

PC9-T790M and H1975 cells were plated in 96-well plates at a density of 350 cells/well and treated with osimertinib and/or T-DM1 following the indicated schedules in the experiment. Medium with drugs was changed every 7 days. Wells were followed daily over time. Taking into account the different sensitivity to osimertinib, cells were scored as resistant when colonies with at least 20 cells were detected in PC9-T790M cell cultures or when >80% confluence was reached in H1975 cell cultures [25].

### Isolation and culture of NK cells and ADCC assay

Highly purified CD56+ natural killer (NK) cells were obtained by magnetic separation and ADCC was measured with the CytoTox 96 non-radioactive cytotoxicity assay (Promega, Madison, WI) as previously described [14].

### Western blot analysis

Procedures for protein extraction, solubilization, and protein analysis by 1-D PAGE are described elsewhere [21]. Antibodies against, EGFR, p-EGFR ^Tyr1068^, HER-2 (29D8), p-HER2 ^Tyr1221/1222^ (6B12), AKT, pAKT^ser473^, p-p44/42 MAPK (D13.14.4E), MAPK (137F5), Bim (34C5) and Mcl-1 (RC-13) were from Cell Signaling Technology (Beverly, MA); antibody against Actin was from Sigma. HRP-conjugated secondary antibodies were from Pierce (Rockford, IL) and chemoluminescence system (Immobilion™ Western Chemiluminescent HRP Substrate) was from Millipore (Temecula, CA). Reagents for electrophoresis and blotting analysis were from BIO-RAD (Hercules, CA). The chemiluminescent signal was acquired by C-DiGit® Blot Scanner and the spots were quantified by Image Studio™ Software, LI-COR Biotechnology (Lincoln, NE).

### Tumor xenografts

Two hundred microliters of matrigel (BD Biosciences) and sterile PBS (1:1) containing 5 × 10^6^ PC9/HER2c1 cells were subcutaneously injected on the flank of Balb/c-Nude female mice (Charles River Laboratories, Calco, Italy). The animals were housed in a protected unit for immunodeficient animals with 12-h light/dark cycles and provided with sterilized food and water *ad libitum*. When tumor volume reached an average size of 200 mm^3^, animals were randomly allocated into four groups (*n* = 8 tumors per treatment group): control, osimertinib, T-DM1 and osimertinib combined with T-DM1. Osimertinib (10 mg/kg in 1% Tween 80) was given once per day, five times per week, by oral gavage. T-DM1 (15 mg/kg in 0.9% NaCl) was given intraperitoneally twice per week. Tumors were measured as previously described [13]. Animals were treated for 2 weeks. Throughout all the experimental period, besides tumor size, animal body weight, posture and gait were monitored. At the end of the experiments, mice were euthanized by cervical dislocation and tumors weighted. All experiments involving animals and their care were performed with the approval of the Local Ethical Committee of University of Parma (Organismo per la Protezione e il Benessere degli Animali) and by the Italian Ministry of Health, in accordance with the institutional guidelines that are in compliance with national (D.Lgs. 26/2014) and international (Directive 2010/63/EU) laws and policies.

### Statistical analysis

Statistical analyses were carried out using GraphPad Prism version 6.0 software (GraphPad Software Inc., San Diego, CA). Results are expressed as mean values ±standard deviations (SD) for the indicated number of independent measurements. Differences between the mean values recorded for different experimental conditions were evaluated by Student’s t-test or by one-way ANOVA followed by Bonferroni’s post-test, and *P* values are indicated where appropriate in the figures and in their legends. P values <0.05 were considered as significant. For in vivo studies comparison among groups was made using two-way repeated measures ANOVA followed by Bonferroni’s post-test (to adjust for multiple comparisons). Adjusted P values of less than 0.05 were considered significant.

## Results

### Osimertinib increases the expression of HER-2 on the cell surface of EGFR-mutated NSCLC cell lines

We first evaluated the effect of osimertinib on total EGFR and HER-2 protein levels in PC9 (E746-A750 deletion) and PC9-T790M (E746-A750 deletion and T790M mutation, generated in our laboratory) [15] cell lines. Both the cell lines were very sensitive to the drug, with IC_50_ of 14.4 ± 2.3 and 7.6 ± 0.5 nM for PC9 and PC9-T790M, respectively.

**Fig. 1:**
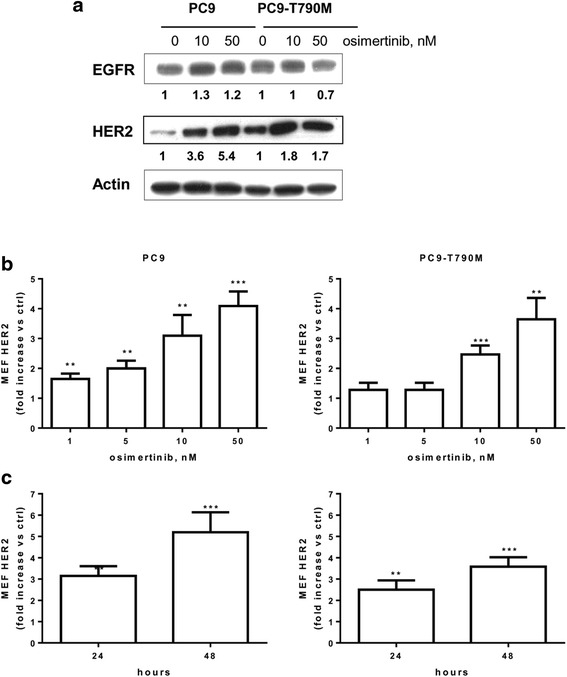
Osimertinib induces cell surface expression of HER-2. **a** PC9 and PC9-T790M cells were treated with the indicated concentrations of osimertinib for 24 h, then cell lysates were immunoblotted to detect the indicated proteins. The immunoreactive spots were quantified by densitometric analysis, ratios of EGFR/Actin and HER2/Actin were calculated and values, expressed as fold increase versus control (control value = 1), are reported. Results are representative of two independent experiments. PC9 and PC9-T790M cell lines were treated with the indicated concentrations of osimertinib for 24 h (**b**) or with 30 nM osimertinib for 24 and 48 h (**c**), then HER-2 protein levels on cell surface was evaluated by flow-cytometry, quantified as MEF, and expressed as fold increase versus control (control value = 1). Mean values of three independent measurements (±SD) are shown (**p < 0.01, ***p < 0.001 versus control; one-way analysis of variance followed by Bonferroni’s post-test)

As shown in Fig. 1a, osimertinib induced a modest increase in the total expression of EGFR protein only in PC9; by contrast, a significant increase in the expression of HER-2 protein was observed both in PC9 and PC9-T790M cells. The levels of EGFR on the plasma membrane, quantified by flow cytometry, was not significantly up-regulated after treatment with osimertinib (not shown). In contrast, osimertinib enhanced HER-2 cell membrane expression in both PC9 and PC9-T790M cells in a dose- (Fig. 1b) and time- (Fig. 1c) dependent manner.

### Effect of osimertinib and T-DM1 combined treatment on cell growth, cell death, signal transduction and antibody-dependent cell-mediated cytotoxicity (ADCC)

**Fig. 2:**
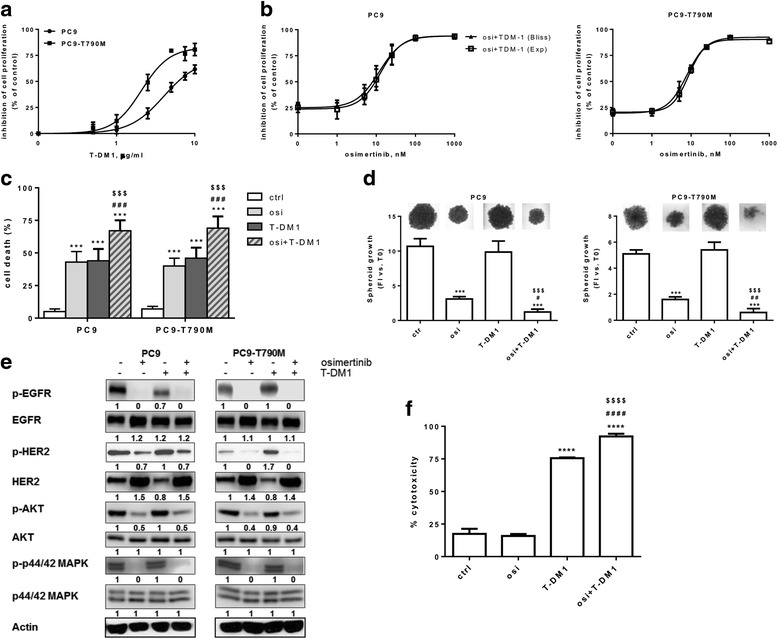
Effects of the combined treatment of osimertinib and T-DM1 on 2D and 3D cell growth, cell death, signal transduction pathways and ADCC. **a** PC9 and PC9-T790M cells were treated with increasing concentrations of T-DM1 for 72 h and then cell proliferation was assessed using crystal violet staining. **b** PC9 and PC9-T790M cells were treated with increasing concentrations of osimertinib in absence or presence of T-DM1 2.5 μg/ml or 1.5 μg/ml, respectively. After 72 h cell proliferation was assessed by MTT assay and the effect of drug combination was evaluated using the Bliss interaction model. Data in **a** e **b** are expressed as percent inhibition of cell proliferation versus control cells and are means ±SD of three separate experiments. **c** cells were treated with the drugs (osimertinib 30 nM, T-DM1 5 μg/ml in PC9, 2.5 μg/ml in PC9-T790M) for 72 h and then cell death was quantitated by fluorescence microscopy analysis on Hoechst 33342 and propidium iodide-stained cells. Data, expressed as percent values, are means ±SD of three independent experiments. **d** spheroids from PC9 and PC9-T790M cells were treated with 30 nM osimertinib, 5 μg/ml (PC9) 2.5 μg/ml (PC9-T790M), T-DM1 or with both drugs and the fold increase (FI) versus T0 was calculated after 6 days of treatment. Representative images of tumor spheroids are shown. Data are from a representative experiment. The experiment, repeated twice, yielded similar results. **e** PC9 and PC9-T790M cells were treated with 30 nM osimertinib in absence or in presence of T-DM1 5 μg/ml or 2.5 μg/ml respectively, and after 6 h the cells were lysed and Western blot analysis was performed to detect the indicated proteins. The immunoreactive spots were quantified by densitometric analysis, ratios versus Actin were calculated and values, expressed as fold increase versus control (control value = 1) are reported. **f** PC9-T790M cells were treated with 30 nM osimertinib for 24 h, then 2.5 μg/ml T-DM1 was added to tumor cells (in absence or in presence of osimertinib) seeded with activated-NK cells at the ratio of 1:25. After 4 h LDH release was determined as described in the Methods section. Data in **e** and **f** are representative of two independent experiments. (***p < 0.001, ****p < 0.0001 vs ctrl; #*p* < 0.05, ##*p* < 0.01, ###p < 0.001, ####p < 0.0001 vs osimertinib; $$$ < 0.001, $$$$ < 0.0001 vs T-DM1; one-way ANOVA followed by Bonferroni’s post-test)

Based on these previous analyses, with the aim to test the efficacy of a combined treatment of osimertinib with trastuzumab or trastuzumab emtansine (T-DM1) in PC9 and PC9-T790M cells, we firstly evaluated the effect of trastuzumab and T-DM1 alone on cell growth. Trastuzumab was ineffective (not shown), while T-DM1 showed anti-proliferative effects with IC_50_ of about 5 μg/ml in PC9 and 2.5 μg/ml in PC9-T790M (Fig. 2a). The higher sensitivity of PC9-T790M in respect to PC9 to T-DM1 may be related to the basal higher expression of HER-2 (see Fig.1a).

We then studied the effect of the association of osimertinib with T-DM1. As shown in Fig. 2b, an additive inhibitory effect on cell proliferation (evaluated by the Bliss interaction model as described in Methods section) was observed in both the cell models. Moreover, the drug combination enhanced the percentage of cell death in respect to single treatments (Fig. 2c). 3D spheroids were developed from PC9 and PC9-T790M cells and their volume was strongly reduced by osimertinib (Fig. 2d). T-DM1 did not inhibit spheroid growth, however in both the cell models the combined treatment of osimertinib with T-DM1 significantly reduced 3D growth in respect to osimertinib alone. EGFR and HER-2 phosphorylation as well as MAPK and AKT signaling transduction pathways were effectively inhibited by osimertinib as expected, being these models very sensitive to osimertinib; T-DM1 induced an increase in HER-2 phosphorylation at 6 h only in PC9-T790M. Nevertheless, the combination of osimertinib and T-DM1 did not differ from the treatment with osimertinib alone in both the cell lines (Fig. 2e). We also examined whether the capability to activate natural killer (NK)-mediated ADCC was enhanced by osimertinib exposure. As shown in Fig. 2f, T-DM1-dependent cytotoxicity in the presence of IL-2-activated NK cells was significantly enhanced in PC9-T790M cells pre-treated with osimertinib, suggesting that the drug may improve the efficacy of T-DM1 in vivo.

### Effect of different combinatory schedules of osimertinib and T-DM1 on cell growth, cell death, and acquisition of osimertinib resistance

**Fig. 3:**
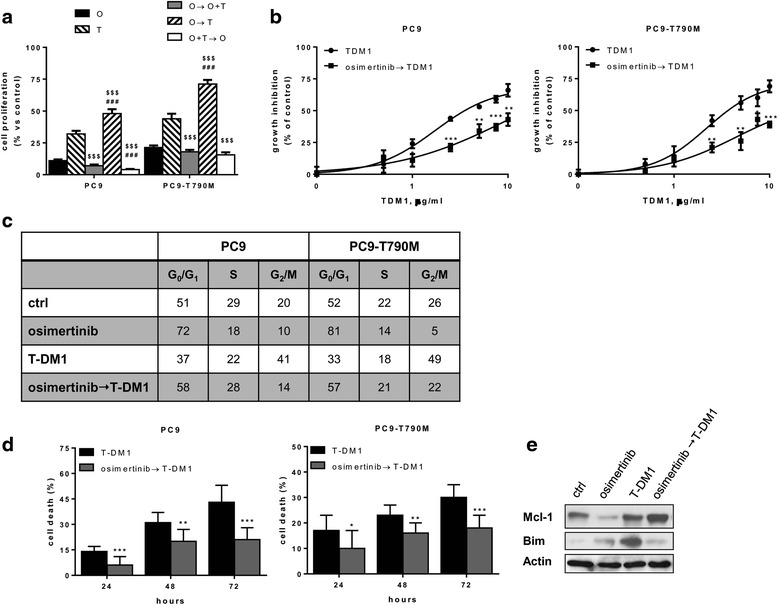
Therapeutic potential of combining osimertinib and T-DM1 using different schedules of administration. **a** PC9 and PC9-T790M cells were treated with osimertinib (30 nM) (O) or T-DM1 (5 μg/ml in PC9 cells and 2.5 μg/ml in PC9-T790M cells) (T) on the basis of the following schedules: 72 h O and then 72 h O plus T (O → O + T); 72 h O and then 72 h T (O → T); 72 h O plus T and then 72 h O (O + T→O). Cell proliferation was assessed by crystal violet assay expressed as % versus control cells (###*p* < 0.001 vs osimertinib; $$$ < 0.001, vs T-DM1; one-way ANOVA followed by Bonferroni’s post-test). **b** cells were treated with increasing concentrations of T-DM1 for 72 h or with osimertinib for 72 h and then with T-DM1 for 72 h. Cell proliferation was assessed using crystal violet staining. Data are expressed as percent inhibition of cell proliferation versus control cells and are means ±SD of three separate experiments (*p < 0.05, ** < 0.01, ***p < 0.001, vs T-DM1; Student’s *t* test). **c** cells were treated with osimertinib for 72 h, T-DM1 for 48 h, or osimertinib for 72 h and then T-DM1 for 48 h, and analyzed by flow cytometry for cell cycle phase distribution. **d** cells were treated with osimertinib for 72 h and then with T-DM1, or with T-DM1 alone and at the indicated times cell death was quantitated by fluorescence microscopy analysis on Hoechst 33,342 and propidium iodide-stained cells. Data are expressed as percent values. (*p < 0.05, ** < 0.01, ***p < 0.001, vs T-DM1; Student’s *t* test). **e** PC9-T790M cells were treated with osimertinib for 72 h, or with T-DM1 for 48 h, or with osimertinib for 72 h and then with T-DM1 for 48 h, cells were lysed and Western blot analysis was performed on lysate proteins by using monoclonal antibodies directed to the indicated proteins. The results in **a**, **c**, **d**, **e** are representative of at least two separate experiments

We then explored the therapeutic potential of combining osimertinib and T-DM1 using different schedules of administration in 72 h + 72 h experiments in PC9 and PC9-T790M. The cells were treated with the following protocols: cells treated only with osimertinib (O) or T-DM1 (T); cells treated for 72 h with osimertinib and then for 72 h with osimertinib plus T-DM1 (O → O + T); cells treated for 72 h with osimertinib and then for 72 h with T-DM1 (O → T); cells treated for 72 h with osimertinib plus T-DM1 and then for 72 h with osimertinib alone (O + T → O). As expected, osimertinib strongly reduced cell growth but the maximum of inhibition in this short-term growth assay was observed when the combined treatment was the first treatment in both the cell models (Fig. 3a). By contrast, a significant number of cells was detected in the protocol “osimertinib intercalated with T-DM1” (O → T) revealing an antagonist effect of osimertinib on the efficacy of T-DM1. Indeed, a pre-treatment with osimertinib significantly reduced the sensitivity to T-DM1 in both PC9 and PC9-T790M increasing the IC_50_ value above 10 μg/ml (Fig. 3b). We also evaluated cell cycle distribution (Fig. 3c) and cell death (Fig. 3d, e). When PC9 and PC9-T790M cells were exposed to T-DM1 for 48 h, a G2/M phase arrest was observed. In contrast, when the cells were treated for 72 h with osimertinib before T-DM1 treatment, a cell cycle profile comparable to that of cycling control cells was observed. Moreover, in the cells pre-treated with osimertinib and then treated with T-DM1 for 48 h cell death was significantly reduced in respect to cells treated for 48 h with T-DM1 alone (Fig. 3d) and an up-regulation of the anti-apoptotic protein Mcl-1 and a down-regulation of the pro-apoptotic protein Bim were observed in osimertinib-pretreated cells exposed for 48 h to TDM-1 compared to cells only treated for 48 h with T-DM1 (Fig. 3e).

These results indicate that an intermittent protocol in which osimertinib is the first treatment and the antibody-chemotherapeutic drug conjugate is the second therapy is not recommended, considering the antagonist effect on cell proliferation and cell death.

**Fig. 4:**
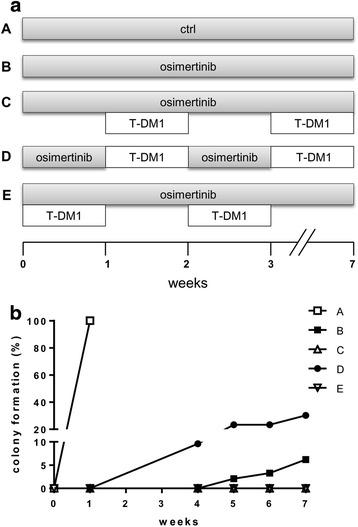
Effect of different schedules of osimertinib and T-DM1 association in the acquisition of osimertinib resistance. Following the schedules indicated in **a,** PC9-T790M cells were plated in 96-well plates at a density of 350 cells/well and treated with osimertinib 30 nM and/or 2.5 μg/ml T-DM1. Cells were scored as resistant when colonies with at least 20 cells were detected (**b**). Results are representative of two independent experiments

We then explored the therapeutic potential of combining osimertinib and T-DM1 using different schedules of administration in a long-term growth assay where PC9-T790M cells were seeded in 96-well plates at low density and treated with different schedules until resistant clones emerged. In particular, cells were cultured following the protocols indicated in Fig. 4a: schedule A (control, ctrl); schedule B (cells treated continuously with osimertinib), schedule C (cells treated continuously with osimertinib and, every 7 days, a simultaneous treatment with T-DM1 was cyclically performed); schedule D (cells treated with osimertinib intercalated, every 7 days, with T-DM1); schedule E (cells treated with osimertinib plus T-DM1 intercalated, every 7 days, with osimertinib alone).

After 5 weeks we detected one colony with more than 20 cells in two wells (2.1%) in schedule B, and the colony number slightly increased up to six colonies (6.2%) after seven weeks (Fig. 4b). The maximum of colony formation inhibition was observed in schedules C and E, indeed, no colonies appeared throughout the duration of the experiment. Differently, in the schedule D, ten resistant colonies were evident after four weeks (9.6%) and reached the number of 29/96 after seven weeks (30.2%).

Considering the difficulty to obtain resistant clones from PC9-T790M cells, and to give strength to our results, we repeated some of the experiments with the less osimertinib-sensitive T790M mutation-harboring H1975 cell line (IC_50_ for osimertinib 100 nM) [25].

**Fig. 5:**
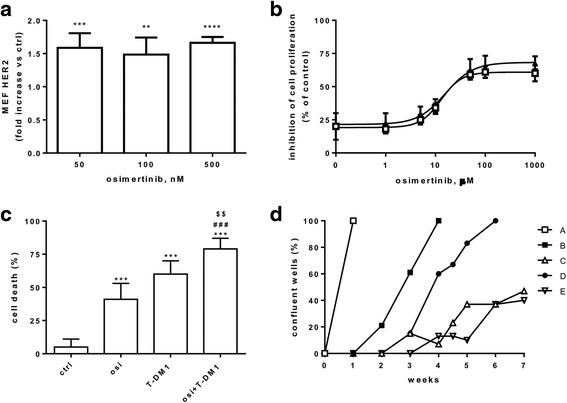
Effects of the combined treatment of osimertinib and T-DM1 on cell growth, cell death and acquisition of osimertinib resistance in H1975 cells. **a** HER-2 protein levels on cell surface were evaluated by flow-cytometry in H1975 cells treated for 48 h with the indicated concentrations of osimertinib, quantified as MEF, and expressed as fold increase versus control. Mean values of three independent measurements (±SD) are shown (**p < 0.01, ***p < 0.001, ***p < 0.0001 versus control; one-way analysis of variance followed by Bonferroni’s post-test). **b** H1975 cells were treated with increasing concentrations of osimertinib in absence or presence of T-DM1 2.5 μg/ml. After 72 h cell proliferation was assessed by MTT assay and the effect of drug combination was evaluated using the Bliss interaction model. Data are expressed as percent inhibition of cell proliferation versus control cells and are means ±SD of three separate experiments. **c** Cells were treated with the drugs (osimertinib 100 nM, T-DM1 5 μg/ml) for 72 h and then cell death was quantitated by fluorescence microscopy analysis on Hoechst 33342 and propidium iodide-stained cells. Data, expressed as percent values, are means ±SD of three independent experiments (***p < 0.001 vs ctrl; ###p < 0.001 vs osimertinib; $$ < 0.01 vs T-DM1; one-way ANOVA followed by Bonferroni’s post-test). **d** Following the schedules indicated in Fig. 4 **a** H1975 cells were plated in 96-well plates at a density of 350 cells/well and treated with osimertinib 100 nM and/or 5 μg/ml T-DM1. H1975 cells were scored as resistant when they had reached >80% confluence. Results are representative of two independent experiments

As shown in fig. 5a, also in H1975 cells osimertinib increased the expression of HER-2 protein, as evaluated by flow cytometry. The effect of the association of osimertinib with T-DM1 (IC_50_ for T-DM1: 5 μg/ml) was then evaluated and an additive inhibitory effect on cell proliferation was documented (Fig. 5b) with a concomitant significant increase in the percentage of cell death, confirming the results obtained in PC9 and PC9-T790M cells (Fig. 5c).

We repeated the long-term culture experiment described in fig. 4a. As expected, we obtained a higher proportion of resistant colonies in a shorter time in respect to PC9-T790M cells and many wells reached confluence (Fig. 5d). In particular, a continuous treatment with osimertinib (schedule B) induced a complete resistance, with 100% of wells (96/96) confluent after 4 weeks; T-DM1 combined with osimertinib intercalated with osimertinib alone (schedules C and E) delayed and reduced the development of osimertinib resistance with less than 50% of confluent wells after seven weeks. By contrast, also in H1975 cells, the schedule D was the less effective in inhibiting colony formation, inducing only a delay of 15 days in reaching 100% of confluence.

### T-DM1 overcomes osimertinib resistance induced by HER-2 overexpression

**Fig. 6:**
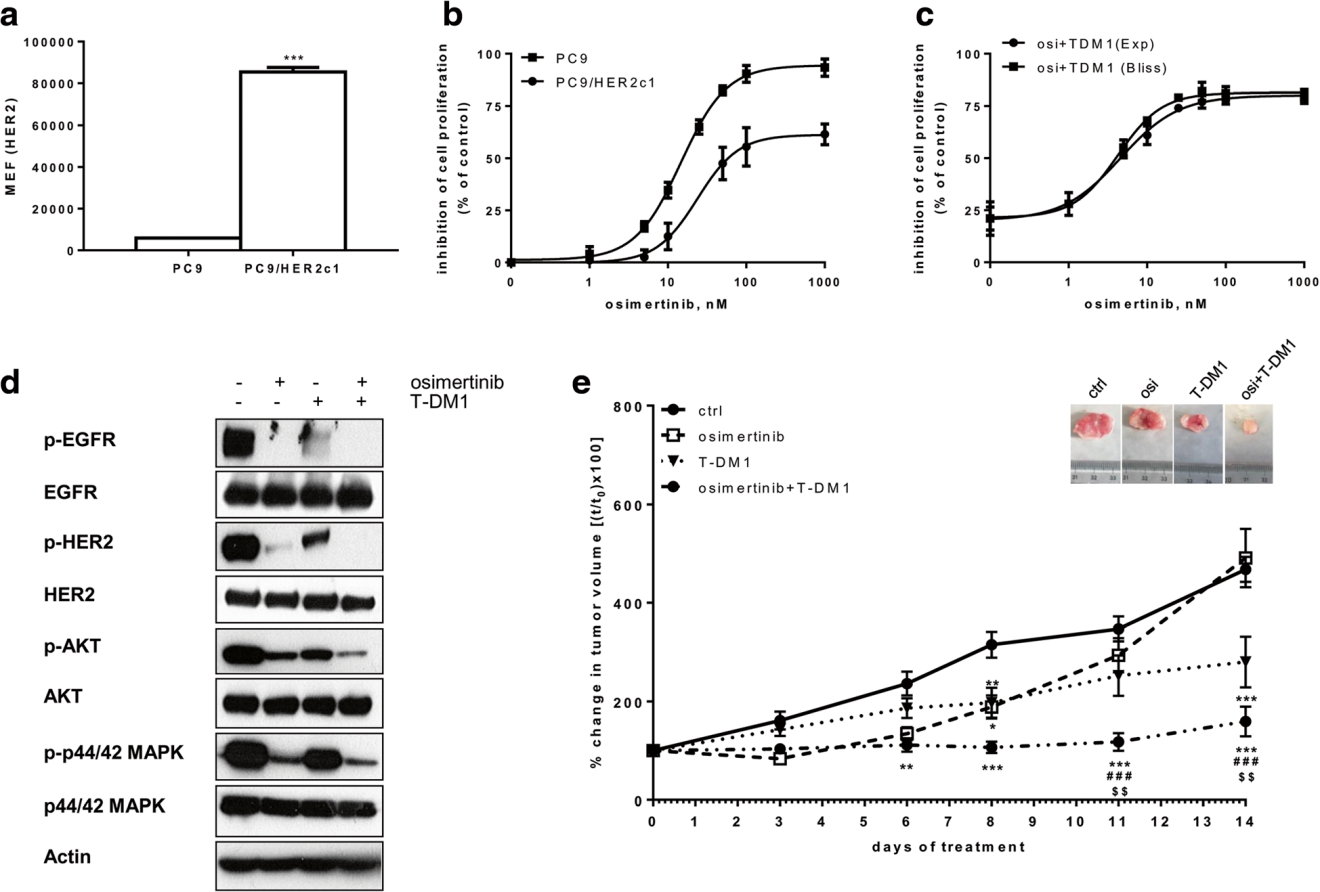
Effect of osimertinib and T-DM1 on HER-2 overexpressing PC9/HER2c1 cells in vitro and in xenograft models. **a** HER-2 protein levels on cell surface was quantified by flow-cytometry and expressed as MEF as described in the Methods section. Mean values of two independent measurements (±SD) are shown (***p < 0.001 versus PC9 cells, Student’s *t* test). **b** PC9 and PC9/HER2c1 cells were treated with increasing concentrations of osimertinib for 72 h and then cell proliferation was assessed using MTT assay. **c** PC9/HER2c1 cells were treated with increasing concentrations of osimertinib in absence or in presence of 1 μg/ml T-DM1. After 72 h cell proliferation was assessed by MTT assay and the effect of drug combination was evaluated using the Bliss interaction model. Data in **b** e **c** are expressed as percent inhibition of cell proliferation versus control cells and are means ±SD of three separate experiments. **d** Cells were treated with 100 nM osimertinib, with 2 μg/ml T-DM1 or with their combination, and after 24 h cells were lysed and Western blot analysis was performed to detect the indicated proteins. **e** PC9/HER2c1 cells were subcutaneously inoculated into Balb/c-Nude female mice and after tumors have reached an average size of about 200 mm^3^ the animals were randomized into four different groups. Osimertinib was administered at a dosage of 10 mg/Kg/die orally and T-DM1 at a dosage of 15 mg/kg twice per week intraperitoneally for 2 weeks. Tumor sizes were measured every 3–4 days and data are expressed as percent change in tumor volume ± SEM (*n* = 8 tumors per treatment group). (* < 0.05, **p < 0.01, ***p < 0.001 vs ctrl; ###p < 0.001 vs osimertinib; $$p < 0.01 vs T-DM1; two-way ANOVA followed by Bonferroni’s post-test)

As recently reported, HER-2 amplification represents a mechanism of resistance to osimertinib in patients [9]. Based on this clinical result, we tested the effect of osimertinib on HER-2 overexpressing PC9/HER2c1 cells (Fig. 6a). HER-2 overexpression significantly reduced the sensitivity to osimertinib (Fig. 6b), confirming previous results [20]. We then tested the effect of combining osimertinib and T-DM1 in this clone. In Fig. 6c the dose-response curve of osimertinib in the presence of a fixed concentration of T-DM1 is shown. Comparing the experimental combination points with the results expected by the Bliss criterion, an additive effect was observed.

Western blot analysis of PC9/HER2c1 cells treated with osimertinib or TDM-1 alone or in combination demonstrated that the combined treatment was the most effective in inhibiting HER-2 phosphorylation and activation of AKT and MAPK pathways (Fig.6d).

To further investigate whether T-DM1 might overcome the intrinsic resistance to osimertinib induced by HER-2 amplification we performed an in vivo experiment. PC9/HER2c1 cells were injected subcutaneously into the flanks of BALB/c nude female mice, and after tumors had reached an average size of about 200 mm^3^, the animals were randomized to receive vehicle (ctrl), osimertinib, T-DM1 or the combined treatment. Osimertinib was administered at a dose of 10 mg/Kg, a dosage able to induce a complete response in PC9-derived xenograft models according to previous studies [3]; T-DM1 was administered at a dose of 15 mg/Kg, as previously reported [13]. Tumor growth was monitored every three-four days for 2 weeks and during this period mice showed no signs of toxicity or other abnormal behavior and regularly gained body weight. As shown in Fig. 6e, osimertinib inhibited tumor growth for 6 days, then tumors grew as the controls indicating resistance to osimertinib; T-DM1 significantly reduced tumor growth, but the combination of osimertinib with T-DM1 completely inhibited tumor growth in comparison with single drug treatments. The efficacy of the combination was well demonstrated by the reduction in volume of tumors excised at sacrifice, compared to control (Inset to Fig. 6e).

## Discussion

In this study we demonstrated the efficacy of T-DM1 when combined with osimertinib in delaying and overcoming resistance to the EGFR TKI in T790M-positive models (PC9-T790M and H1975 cells) and in PC9/HER2 cell lines, respectively.

Osimertinib is an effective treatment in NSCLC patients with T790M EGFR mutation, progressed after first-line therapy with first- or second-generation EGFR-TKIs [4]. Moreover, osimertinib was also evaluated in the first-line setting in patients with EGFR activating mutations compared with gefitinib or erlotinib (FLAURA Trial NCT02296125). In this trial, recently presented at ESMO meeting [26], osimertinib showed a statistically significant PFS benefit vs. gefitinib/erlotinib [median PFS 18.9 months in the osimertinib group (95% CI, 15.2, 21.4) and 10.2 months in the gefitinib/erlotinib group (95% CI, 9.6, 11.1); HR 0.46 (95% CI, 0.37, 0.57), *p*-value <0.0001]. However, also for this agent, resistance is expected and new therapeutic approaches are needed to prolong its benefit [7].

HER-2 was shown to be overexpressed (IHC 3+) in 2–6% of NSCLC, with gene amplification found in 2–4%, mainly in the adenocarcinoma histotype [27]. In particular, HER-2 amplification has been identified as a potential mechanism of osimertinib acquired resistance [9, 20].

We tested T-DM1 in preclinical experiments suggesting its activity against HER-2-driven lung cancer cell lines in vitro [13]. Only recently this agent was evaluated in patients with HER2-driven NSCLC in two trials presented at ASCO meeting in 2017 [28, 29]. Stinchcombe et al. [28] reported primary results from an ongoing phase 2 study (NCT02289833) of 49 patients with previously treated HER2-overexpressing advanced NSCLC who received single-agent T-DM1, showing objective tumor responses only in 4 patients with elevated (3 +) HER2 immunohistochemical expression. In the Li et al. study (NCT02675829) [29] patients with HER2-mutant lung cancers were enrolled into a cohort of the basket trial of TDM1 in HER2-amplified or mutant cancers. This study has met its primary endpoint, evidencing a tumor response in 8 out of 18 (44%) patients, across different mutation subtypes.

As previously reported by Scaltriti et al. for lapatinib in breast cancer cells [30] and by our group for erlotinib in EGFR wild-type NSCLC cells [14], we demonstrated, in this study, that also osimertinib increased the cell surface expression of HER-2 in EGFR-mutated PC9 in PC9-T790M and in H1975 cell lines. The efficacy of dual inhibition of EGFR and HER-2 has been documented in EGFR wild-type NSCLC cell lines combining gefitinib or erlotinib with trastuzumab or pertuzumab [14, 31, 32], and in EGFR T790M mutant cell lines combining anti-EGFR, anti HER-2 and anti HER-3 antibodies [33]. This combination strategy has not been evaluated yet in NSCLC clinical trials, probably considering the availability of agents able to block EGFR and HER-2 simultaneously, such as afatinib and dacomitinib [18].

Here we demonstrated that, differently from trastuzumab, T-DM1 showed anti-proliferative effects in PC9, PC9-T790M and H1975 cells and exerted an additive effect when combined with osimertinib in terms of inhibition of proliferation, cell death and ADCC induction. Because osimertinib is highly effective in mutated NSCLC cell lines, the addition of T-DM1 caused a modest benefit when evaluated in short-term growth assay, by contrast the combination significantly delayed the acquired resistance to osimertinib in PC9-T790M and in the less osimertinib-sensitive model H1975. The combination was effective either when the cells were treated continuously with osimertinib and a simultaneous treatment with TDM1 was cyclically performed (see Fig. 4 and Fig. 5, schedule C) or when the cells were treated with osimertinib plus TDM1 intercalated with osimertinib alone (schedule E). It is worthy of note that the application of an intermittent treatment, in which osimertinib was switched with T-DM1 (schedule D), demonstrated a negative interaction between osimertinib and T-DM1 with a strong reduction in sensitivity to T-DM1 in both PC9 and PC9-T790M cells. In addition, an up-regulation of the anti-apoptotic Mcl1 protein and a down-regulation of the pro-apoptotic Bim protein were documented in cells treated with osimertinib and then exposed to T-DM1 compared to cells treated with T-DM1 alone. Similarly, a drug-antagonism has been also demonstrated when a EGFR-TKI was administered before pemetrexed [15, 34].

At present, to our knowledge, this is the first study demonstrating that osimertinib reduces the efficacy of an antibody-chemotherapeutic drug conjugate given as second treatment; moreover, the novelty of this study is related to the effect of different combinatory schedules in the acquisition of osimertinib resistance. In PC9-T790M cells, the discontinuous protocol (osimertinib→T-DM1) anticipated the appearance of resistance to osimertinib compared to cells continuously treated with osimertinib alone, by contrast in the protocols in which cells were simultaneously exposed to both drugs (osimertinib→osimertinib + T-DM1 or osimertinib + T-DM1→osimertinib), no resistant colonies were detected up to 50 days of treatment. In H1975 cells, all schedules delayed the acquisition of resistance; however, also in this model the less effective was the intermittent schedule (osimertinib→T-DM1) both in terms of percentage of resistant wells and of median time to resistance.

Overall, these data may support the use of a combination of osimertinib with T-DM1 using a specific schedule to delay or prevent the acquisition of resistance to the EGFR-TKI, similarly to other combinations of third-generation EGFR-TKIs (with MEK or MET inhibitors) tested in clinical trials currently recruiting patients [7].

In the last part of this study we investigated the role of HER-2 amplification in the responsiveness to osimertinib and the efficacy of T-DM1 in vitro and in vivo to overcome osimertinib resistance. HER-2 amplification, indeed, has been reported as a mechanism of acquired resistance to EGFR-TKIs with a frequency of 12–13% [2, 35].

PC9/HER2c1 cells were exposed to osimertinib in vitro and in vivo in xenotransplanted mice. These cells were significantly more resistant to osimertinib than the parental cells in vitro and tumors grew as the controls indicating intrinsic resistance to osimertinib. T-DM1 significantly reduced tumor growth but the combination of osimertinib with T-DM1 showed a complete suppression of tumor growth, suggesting an important role of T-DM1 in overcoming osimertinib resistance. These data confirm the role of HER-2 amplification as a potential cause of intrinsic resistance to osimertinib, as demonstrated by Ortiz-Cuaran S et al. [20], and indicate the combination with TDM1 as a potential strategy to overcome such resistance. Although in the absence of a cell line model with EGFR-activating/T790M developing HER-2 amplification as a resistance mechanism, these results are particularly relevant considering the potential future use of osimertinib in first-line therapy for EGFR-mutated NSCLC patients.

## Conclusions

In conclusion, the combination of osimertinib with T-DM1 exerted an additive effect on cell growth inhibition when given as a concomitant administration. However, when cells exposed to osimertinib were subsequently treated with T-DM1, an antagonist interaction was observed. The combination of T-DM1 with osimertinib retarded the appearance of resistance in PC9-T790M cells and overcome osimertinib-resistance in cells with HER-2 amplification. Our results may contribute to define new potential future drug-combinations in the treatment of EGFR mutated NSCLC patients.

## Abbreviations

EGFR: Epidermal Growth Factor Receptor
MEF: Molecules of Equivalent Fluorochrome
NSCLC: Non-Small Cell Lung Cancer
T-DM1: Trastuzumab Emtansine
TKIs: Tyrosine Kinase Inhibitors
